# Establishment of Complex Modulus Master Curves Based on Generalized Sigmoidal Model for Freeze–Thaw Resistance Evaluation of Basalt Fiber-Modified Asphalt Mixtures

**DOI:** 10.3390/polym12081698

**Published:** 2020-07-29

**Authors:** Guojin Tan, Wensheng Wang, Yongchun Cheng, Yong Wang, Zhiqing Zhu

**Affiliations:** 1 College of Transportation, Jilin University, Changchun 130025, China; tgj@jlu.edu.cn (G.T.); chengyc@jlu.edu.cn (Y.C.); zhuzqjlu@163.com (Z.Z.); 2 Jilin Province Highway Administration Bureau, Changchun 130021, China; wangyjlgl@gmail.com

**Keywords:** asphalt mixture, basalt fiber, freeze–thaw cycle, complex modulus, generalized Sigmoidal model

## Abstract

This study aims to study the freeze–thaw (F–T) resistance of asphalt mixture incorporating styrene–butadiene–styrene (SBS) polymer and basalt fiber by using the established complex master curves of the generalized Sigmoidal model. Asphalt mixture samples incorporating styrene–butadiene–styrene (SBS) polymer and basalt fiber were manufactured following the Superpave gyratory compaction (SGC) method and coring as well as sawing. After 0–21 F–T cycles processing, a complex modulus test asphalt mixture specimen was performed to evaluate the influence of the F–T cycle. Besides, according to the time–temperature superposition principle, the master curves of a complex modulus were constructed to reflect the dynamic mechanical response in an extended range of reduced frequency at an arbitrary temperature. The results indicated that the elastic and viscous portions of asphalt mixture incorporating SBS and basalt fiber have decreased overall. It could be observed from the dynamic modulus ratio that the dynamic modulus ratios of specimens were more affected by the F–T cycle at low frequency or high temperature. Thus, in the process of asphalt pavement design and maintenance, attention should be paid to seasonal frozen asphalt pavement under low frequency and high temperature.

## 1. Introduction

As an important part of transportation infrastructure, asphalt pavement plays a significant role in social development. With the increase of traffic demand, ordinary asphalt pavements often fail to meet the performance requirements, resulting in a few destructions including low and medium-temperature cracking, high-temperature rutting and freeze–thaw (F–T) destruction, and so on [1,2,3,4]. To improve mechanical performances, various additives such as rubbers, polymers, fibers, and other additive materials have been adopted to incorporate with asphalt [5,6,7,8]. Studies demonstrated that polymers including styrene-butadiene-styrene (SBS), styrene-butadiene-rubber (SBR), etc. have been proved to improve the high-temperature rutting, moisture damage, and so on [9]. Besides, adding fibers to asphalt mixtures usually increases the mechanical performances such as cracking resistance [10]. To improve the compressive capabilities of asphalt materials effectively, researchers have made lots of efforts and tried many novel additives.

SBS polymer has been proven to improve asphalt well due to its dual characteristics of rubber and plastic, and most published studies have investigated SBS-modified asphalt systematically [11,12,13]. Today, the SBS polymer modifiers are commonly applied in modified bitumen, and the demand for SBS modifiers is increasing with the development of highway construction. Imaninasab [14] investigated and evaluated the influences of the modification process of two kinds of polymers, i.e., SBS and granular polymers including Rheofalt and Lucobit, on anti-rutting performance at a high temperature of stone mastic asphalt (SMA). Test results indicated that the direct modification process of polymers added into asphalt mixtures was not as efficient as the modification process of polymer-modified asphalt into mixtures. Besides, it was indicated that the elastic modulus of bituminous mixes would reduce as the SBS content increased. Wang et al. [15] explored the experimental methods of polymer-modified asphalt (PMA) including SBS with different proportions systematically so as to ensure the quality and requirements of construction engineering in asphalt pavement. The results showed that PMA could be evaluated effectively using indirect tensile strength and the phase angle, but the rolling-thin film oven (RTFO) did not reflect the real field aging condition of PMA pavement. Hajikarimi et al. [16] investigated the rheological and mechanical performances of SBS-modified bitumen as well as its binder with three proportions by using rheometer equipment for the purpose of analyzing the storage and loss modulus and viscoelastic behavior. Furthermore, they used the obtained dynamic test results in the finite element software ABAQUS to simulate the viscoelastic behavior of neat and SBS-modified asphalt, respectively. The optical microscope picture was also employed to eliminate the error of numerical modeling [17].

As a novel environmentally friendly mineral fiber, basalt fiber has several excellent advantages including better strength and high temperature, as well as acid and alkali resistance [18]. In recent years, basalt fiber has gained more and more attention for improving asphalt mixture, and many scholars have been devoted to studying the impact of basalt fiber on the performance improvement of bituminous materials [19,20]. Studies demonstrated that basalt fibers have a greater impact on enhancing the comprehensive performance of bituminous materials to some extent. Sun et al. [21] explored an enhancement impact of basalt fiber on the toughness of bituminous materials. The results showed that the asphalt mixture with a fiber content of 0.4% by weight would be better. Qin et al. [22] investigated the influences of basalt fibers with various sizes and contents on bituminous mastics and compared them with other common fibers such as lignin fiber and polyester fiber. They drew a conclusion that basalt fiber has the best comprehensive performances, and basalt fiber with 6 mm was suggested due to its larger contact area with asphalt. Li et al. [23] conducted three-point bending tests at three low temperatures on asphalt concretes (AC-13 and AC-20) with various basalt fiber contents and proposed a distinction method of fracture type based on the bending coefficient. They also analyzed the low-temperature improvement mechanism of basalt fiber on asphalt mixture via scanning electron microscopy.

In view of the good improvement effects of SBS and basalt fiber on different aspects of asphalt, most researchers tried to employ the incorporation of polymers and fibers into asphalt mixture to improve its comprehensive performance. Gu et al. [24] pointed out that SBS-modified bituminous reinforced with basalt fiber has a higher rutting factor compared with original asphalt based on dynamic shear rheological tests and the repeated creep tests. It was reported that the high-temperature improvement effect of basalt fiber was significant compared with commonly used fibers. Tanzadeh et al. [25] investigated the open-graded friction course (OGFC) modified by polymer and basalt fiber by the drainage test and common mechanical test. Test results revealed that OGFC with 0.2% basalt fiber and 4.5% SBS polymer had better performances and basalt fiber had a positive effect on reducing the draindown phenomenon. Miao et al. [26] examined four types of fibers (i.e., fiber-reinforced plastic, two lignin fibers, and basalt fiber) and four types of asphalt including neat asphalt and polymers-modified asphalt based on the interfacial properties. The results indicated that basalt fiber had the prime reinforcement, and SBS-modified asphalt was found to be well reinforced with fiber. Luo et al. [27] evaluated the enhancement impact of SBS and basalt fiber on the anti-rutting and anti-cracking of modified asphalt mixture by the Hamburg wheel track test and low-temperature bending test, respectively. Kou et al. [28] selected basalt fiber to reinforce SBS-modified asphalt, and they found that SBS-modified asphalt together with basalt fiber can make use of both advantages of additives.

In order to better apply asphalt mixture to the northeast seasonal frozen regions, a series of studies have been carried out toward exploring the F–T destruction characteristics of bituminous materials [29,30,31,32]. In addition, many scholars conducted various experiments of bituminous mixes under varying F–T actions to explore the damage evolution of bituminous materials [33,34,35]. Tarefder et al. [36] analyzed the influences of F–T action on bituminous materials using several experimental methods on fatigue and rheometer. Test results showed that the fatigue life and creep stiffness of bituminous materials decreased due to the action of F–T cycles. For asphalt mixture with basalt fiber incorporating with SBS and fiber, Liang et al. [37] investigated its fracture characteristics and analyzed the mechanical performance under the action of F–T based on the acoustic emission method. Fan et al. [38] aimed at the quantitative evaluation of fatigue performance of bituminous materials under the repeated F–T action. Cheng et al. [39] made an overall assessment of the mechanical properties of bituminous mixes with basalt fiber and analyzed the improvement impact of F–T resistance based on volumetric and mechanical parameters. Furthermore, they analyzed logistic F–T damage models of bituminous mixes and established a multi-variable gray model [40]. Cheng et al. [41] established a damage evolution of the mechanical performance of bituminous mixes exposed to repeated F–T actions through reliability and damage theory and predicted and analyzed its internal damage degradation. Badeli et al. [42] explored the influences of F–T actions on the fatigue cracking of bituminous mixes considering seasonal ambient temperature variations. Eric et al. [43] evaluated the moisture stability and performance degradation of bituminous mixes subjected to F–T action and studied the viscoelastic behavior of asphalt mixture.

According to the above studies, it is known that there are many studies on the performance of SBS polymer-modified asphalt mixture incorporating basalt fiber and the evaluation of F–T damage of asphalt mixtures. However, at present, most studies focused on the conventional static mechanical response based on the Mechanistic–empirical pavement design guide method but often ignored dynamic mechanical responses (DMA). Hence, this study aims to analyze the dynamic mechanical response for asphalt mixture incorporating SBS polymer and basalt fiber. The master curves of complex modulus were established followed by the generalized Sigmoidal model. To understand the influence of F–T, a dynamic modulus ratio was introduced to evaluate the dynamic mechanical property evolution of bituminous materials incorporating SBS polymer and basalt fiber under varying F–T actions.

## 2. Experimental Procedures

### 2.1. Experimental Materials and Samples

#### 2.1.1. Experimental Materials

SBS-modified asphalt, as one of the most commonly used bitumen types, was selected in this study, which was from Yingkou, China. Its technical parameters are listed in Table 1. Crushed basalts were used as the aggregate type and limestone powder was used as the filler, which were provided by Jiutai City and Siping City, China, respectively. The corresponding technical parameters are summarized in Table 2, Table 3 and Table 4. Then, a novel environmentally friendly mineral fiber, i.e., basalt fiber, was employed as the fiber stabilizer, and the experimental parameters are shown in Table 5.

#### 2.1.2. Samples Preparation

**Figure 1:**
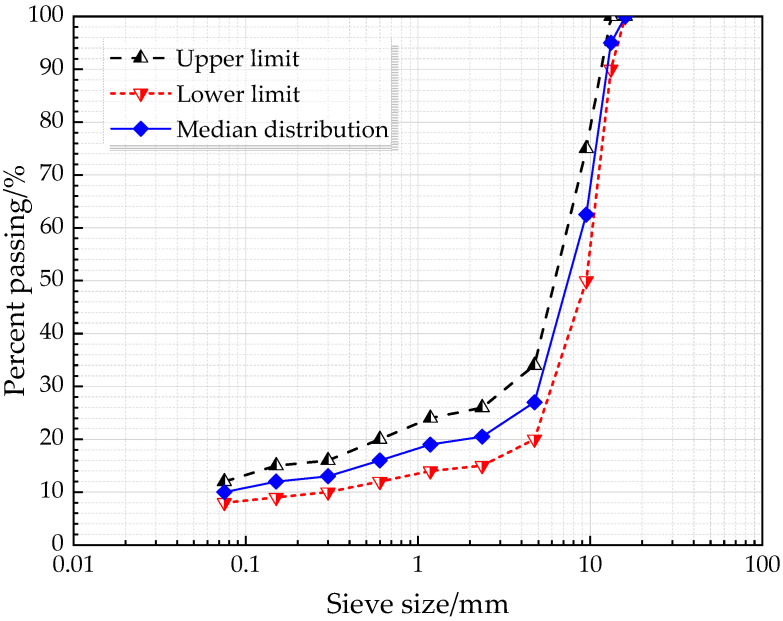
Gradation curve of SMA-13.

The asphalt mixture type SMA was employed in this study. As known, SMA is a widely used asphalt mixture type composed of high-content coarse aggregate, mineral powder, and asphalt as well as low-content fine aggregate, which was initially formed in Germany in the 1960s and first applied in China in 1992 [8]. Due to the better resistance to deformation and durability, SMA has been extensively applied for most of the pavement surfaces of highways in China. The gradation curve of SMA-13 is depicted in Figure 1 and Table 6, in which the median gradation was chosen for manufacturing samples.

The sample preparation procedure followed the Chinese standard JTG E20-2011 [44]. There are several commonly used molding methods, including Marshall compaction, Superpave gyratory compaction (SGC), roller compaction, static pressure molding, and so on. As one of the important technical achievements of the strategic highway research program (SHRP), SGC is a novel Superpave mixture design and molding method. Furthermore, it has been proved that the internal structures of SGC asphalt mixture samples are consistent with core specimens from an actual road with a good correlation, which have less porosity variability.

**Figure 2:**
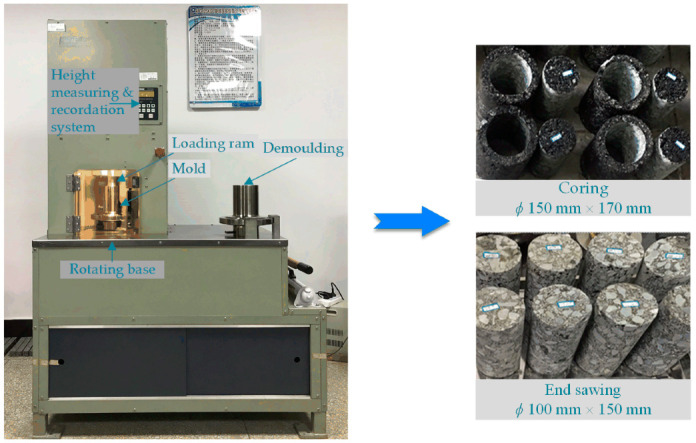
Samples preparation procedure in this study.

In order to better simulate the actual pavement construction, the SGC method was selected to prepare asphalt mixtures with basalt fiber in this study. The detailed procedure of specimen preparation has been described including proportions of basalt fiber and SBS, mixing process, mixing speed and duration, and the set SGC parameters can be found in previous studies [7,8,41]. As shown in Figure 2, asphalt mixture specimens were first manufactured following the SGC method. After de-molding and cooling, asphalt mixture samples (diameter: 100 mm, height: 150 mm) could be prepared through using a core drilling machine and a cutting machine from original samples (diameter: 150 mm, height: 170 mm).

### 2.2. Experimental Procedure and Protocol of Complex Modulus Test

#### 2.2.1. Experimental Procedure

**Figure 3:**
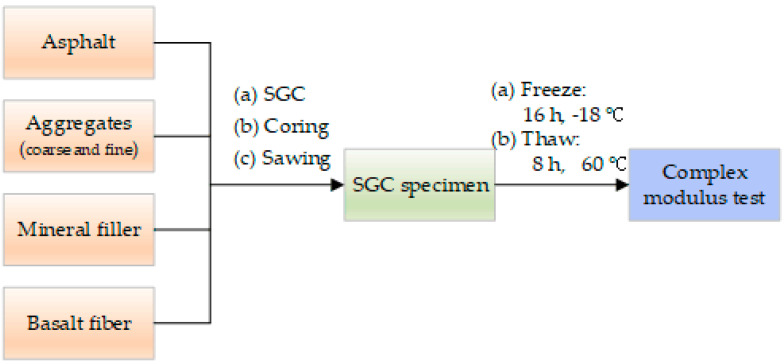
Experimental process flowchart.

Samples of bituminous mixes incorporating SBS polymer and basalt fiber were firstly manufactured and molded following the SGC method in this study. After that, before the performance test, samples were treated by 0–21 F–T cycles, respectively. The detailed procedure of F–T cycle processing for specimens has been described in the previous study [41]. Then, the complex modulus test was conducted under sinusoidal (haversine) compressive loadings at five temperatures and six frequencies from the lowest to highest temperature [45]. The experimental process flowchart is presented in Figure 3.

#### 2.2.2. Protocol of Complex Modulus Test

**Figure 4:**
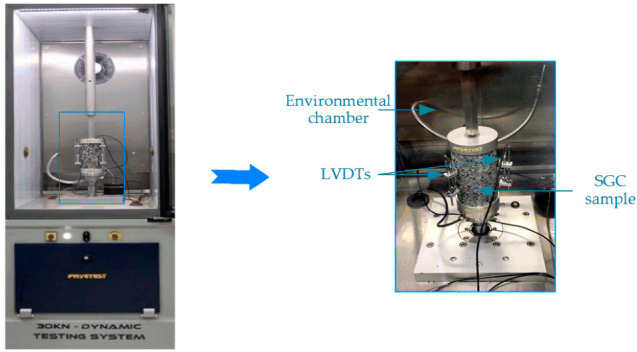
Complex modulus test by dynamic testing system (DTS) in this study.

In the experimental design, the complex modulus test was employed for the dynamic mechanical analysis of asphalt mixture. The complex modulus test is a common dynamic experimental method that is commonly used in asphalt mixture testing. In this experiment, a dynamic testing system (DTS) with a servo-hydraulic actuator of 30 kN and a stroke of 100 mm (DTS-30, MATEST Ltd., Rome, Italy) was employed to perform complex modulus tests according to the specification of AASHTO TP 79 [45]. The DTS with an environmental chamber has a loading range of ±16 kN and a frequency of up to 70 Hz, and it is controlled within −20 to 80 °C. Before the complex modulus test, equipped samples were kept in a testing chamber to equilibrate to the specified condition, as illustrated in Figure 4, in which a monitoring sample was used as a reference. Three linear variable differential transformer (LVDT) brackets were placed and glued to asphalt mixture specimens at three locations 120 degrees apart, as shown in Figure 4.

For bituminous mixes incorporating SBS polymer and basalt fiber under varying F–T actions, the test sample was applied to a haversine compressive force in a cyclic manner during the complex modulus test. The complex modulus tests were also carried out at specific conditions from lower temperature to higher temperature and from higher frequency to lower frequency following the specification of AASHTO TP 79 [45]. Besides, each specimen was tested at five temperatures and six frequencies. There are 24 samples prepared for experimental study. The three vertical LVDTs could record the real-time axial strains of asphalt mixture specimens, which would be adjusted between 70 and 120 microstrain by the DTS automatically. After that, the corresponding mechanical test results could be obtained by the DTS software Testlab [46].

### 2.3. Theory of Viscoelastic Mechanics of Asphalt Mixture

#### 2.3.1. Dynamic Mechanical Response of Viscoelastic Materials

**Figure 5:**
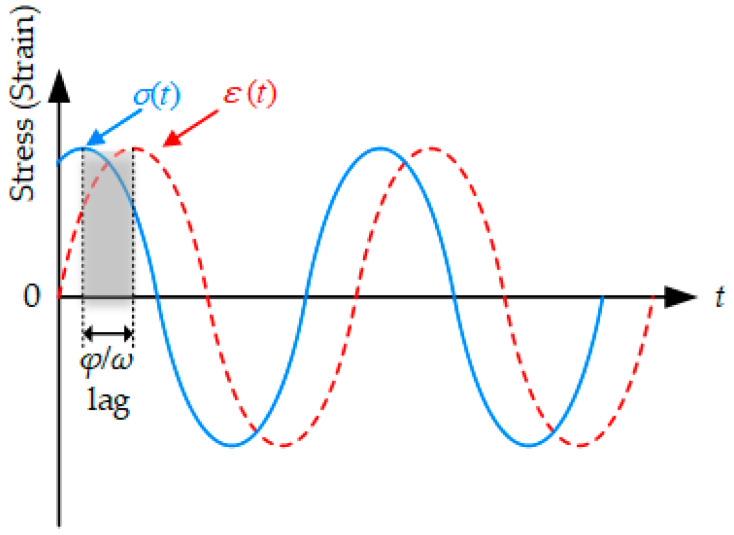
Curves of stress and strain in complex modulus test.

As previously mentioned, DMA is an increasingly important aspect for analyzing the mechanical response of viscoelastic materials, in which the complex modulus test is a commonly used dynamic test method. In traditional asphalt mixture design, static mechanical responses such as compressive resilience modulus are used as indicators. However, the mechanical performances of asphalt pavement in a real environment are affected by environmental factors such as temperature and vehicle load. DMA can effectively reflect the force status of asphalt pavement under repeated vehicle loads in a real environment, which is close to the actual service status. When an asphalt mixture sample is applied to a sinusoidal stress load (or sinusoidal strain load), its corresponding sinusoidal strain response (or sinusoidal stress response) is generally obtained, which is called a stress (or strain) controlled test. For a specimen under an applied cyclically varying sinusoidal load, its strain response would show a cyclically varying trend, but the strain response always lags the applied stress, which is the so-called stress–strain lag phenomenon, as plotted in Figure 5.

In a strain-controlled test, the applied strain-controlled load in a complex plane can be expressed as:

(1) $$\varepsilon (t)=\varepsilon_{0}(\cos\omega t+i\sin\omega t)=\varepsilon_{0}e^{i\omega t}$$

where *ε*_0_ is the strain amplitude, *i* is an imaginary complex number, and *ω* is the angular frequency.

Then, the stress response can be expressed as:

(2) $$\sigma (t)=\sigma^{*}e^{i\omega t}=\sigma_{0}e^{i(\omega t+\varphi )}$$

where σ* is a complex number of stress response amplitude, σ_0_ is an absolute value of complex number σ*, and *φ* is a phase angle.

According to the viscoelastic differential constitutive model, the relationship between E*(*ω*) and *E*(*t*) could be obtained by combining Equations (1) and (2):

(3) $$E^{*}(\omega )=i\omega \bar{E}(i\omega )$$

Equation (3) is expressed as a complex number in its frequency domain:

(4) $$E^{*}(\omega )=E^{\prime }(\omega )+iE^{\prime \prime }(\omega )=\left|E^{*}(\omega )\right|(cos\varphi +i\sin\varphi )=\left|E^{*}(\omega )\right|e^{i\varphi }$$

where the real part $E^{\prime }(\omega )$ of complex modulus E*(*ω*) is the stored energy of viscoelastic materials under alternating stress, i.e., storage modulus, representing the elastic portion; the imaginary part $E^{\prime \prime }(\omega )$ is the energy dissipated as heat, representing the viscous portion. Its absolute value of E*(*ω*), $\left|E^{*}(\omega )\right|=\sqrt{{E^{\prime }}^{2}+{E^{\prime \prime }}^{2}}$, is the dynamic modulus.

The tangent of the phase angle (tan*φ*) between stress and strain can be expressed as follows:

(5) $$\tan\varphi =\frac{E^{\prime \prime }(\omega )}{E^{\prime }(\omega )}$$

The tangent of the phase angle (tan*φ*) provides a measure of damping in the material.

#### 2.3.2. Time–Temperature Equivalence Principle and Construction of Master Curves

The viscoelastic properties of bituminous mixes have an obvious dependence on time and temperature. In the polymer physics, the time–temperature superposition principle can be adopted to analyze the properties at unknown conditions based on known conditions. Then, a master curve at a specified condition would be calculated and adopted to analyze the properties in a larger condition range, which would greatly reduce the test workload.

The determination of shift factor (*α_T_*) is the key to the time–temperature superposition principle. To establish a master curve, a shift factor (*α_T_*) usually needs to be calculated in advance for translating viscoelastic properties at other conditions to a reference experimental condition, and it could be defined as follows:

(6) $$\alpha_{T}=\frac{f}{f_{r}}$$

where *f* is a loading frequency at any temperature, and *f_r_* is the corresponding reduced frequency at a reference temperature.

In general, there are three commonly used time–temperature shift factor equations for determining and establishing the master curve, i.e., the Williams–Landel–Ferry (WLF) equation, Arrhenius equation, and Log-linear equation. In this study, the WLF in the Equation (7) is chosen to calculate the shift factor (*α_T_*).

(7) $$\log\alpha_{T}=-\frac{C_{1}(T-T_{r})}{C_{2}+(T-T_{r})}$$

where *C*_1_ and *C*_2_ are fitting parameters, *T* is test temperature, and *T_r_* is reference temperature.

According to the theory of the time–temperature superposition principle, the viscoelastic properties of bituminous mixes can be analyzed in a wider condition range. In general, the master curve of bituminous materials can be characterized by using the Sigmoidal model, i.e., the S-shaped growth model. However, the standard Sigmoidal model is only applicable to the case in which data points are symmetrical with respect to the turning point of the master curve. Besides, this standard Sigmoidal model ignores the loss modulus and phase angle, resulting in Sigmoidal model being inconsistent with actual test results. Hence, the generalized Sigmoidal model was adopted to establish the master curve of complex modulus of bituminous mixes. The master curve based on the generalized Sigmoidal model can accurately describe the viscoelastic performance of asphalt mixture and provide the elasticity and viscous mechanical behavior of asphalt mixture in a wider range of time and temperatures.

## 3. Results and Discussion

### 3.1. Influence Analysis of Freeze–Thaw Cycles on Dynamic Modulus and Phase Angle of Asphalt Mixture Reinforced with Basalt Fiber

According to the complex modulus test records for the 30 combinations of temperature and frequency, its normal value |E*| and phase angle φ could be derived by Equations (8) and (9).

(8) $$\left|E^{*}\right|=\frac{\sigma_{0}}{\varepsilon_{0}}$$

(9) $$\varphi =\frac{t_{i}}{t_{p}}\times 360$$

where *σ*_0_ and *ε*_0_ are the magnitude of axial stress and strain, respectively; *t_i_* is the average lag time between deformation peak and load peak; and *t_i_* is the average loading period.

The generalized Sigmoidal model expressed in Equation (10) is employed to establish the master curve of dynamic modulus of bituminous mixes.

(10) $$\mathrm{lg}\left|E^{*}\left(f_{r}\right)\right|=\delta +\frac{\alpha -\delta }{{\left(1+\lambda \cdot e^{\beta +\gamma \mathrm{lg}f_{r}}\right)}^{\frac{1}{\lambda }}}$$

where $\mathrm{lg}\left|E^{*}\left(f_{r}\right)\right|$ is the dynamic modulus in logarithmic coordinates, *δ* is the value of the lower asymptote of dynamic modulus |E*|, *α* is the value of the upper asymptote of dynamic modulus |E*|, and *λ*, *β*, and *γ* are shape factors.

Based on linear viscoelastic theory, the two parts expressed in Equation (4) of a complex modulus of bituminous materials usually need to satisfy the Kramers-Kronig (K–K) relationship. According to Equation (10) and the K–K relationship, the semi-log generalized Sigmoidal model can be derived for the master curve of the phase angle, as presented in Equation (11).

(11) $$\varphi \left(f_{r}\right)=-\frac{\pi }{2}\cdot \frac{(\alpha -\delta )\gamma e^{\beta +\gamma \mathrm{lg}f_{r}}}{{\left(1+\lambda \cdot e^{\beta +\gamma \mathrm{lg}f_{r}}\right)}^{\left(1+\frac{1}{\lambda }\right)}}$$

where $\varphi \left(f_{r}\right)$ is the phase angle in logarithmic coordinates, *δ* is the value of the lower asymptote of the phase angle, *α* is the value of the upper asymptote of the phase angle, and *λ*, *β* and *γ* are shape factors.

#### 3.1.1. Construction of Master Curves of Dynamic Modulus under Freeze-Thaw Cycles

**Figure 6:**
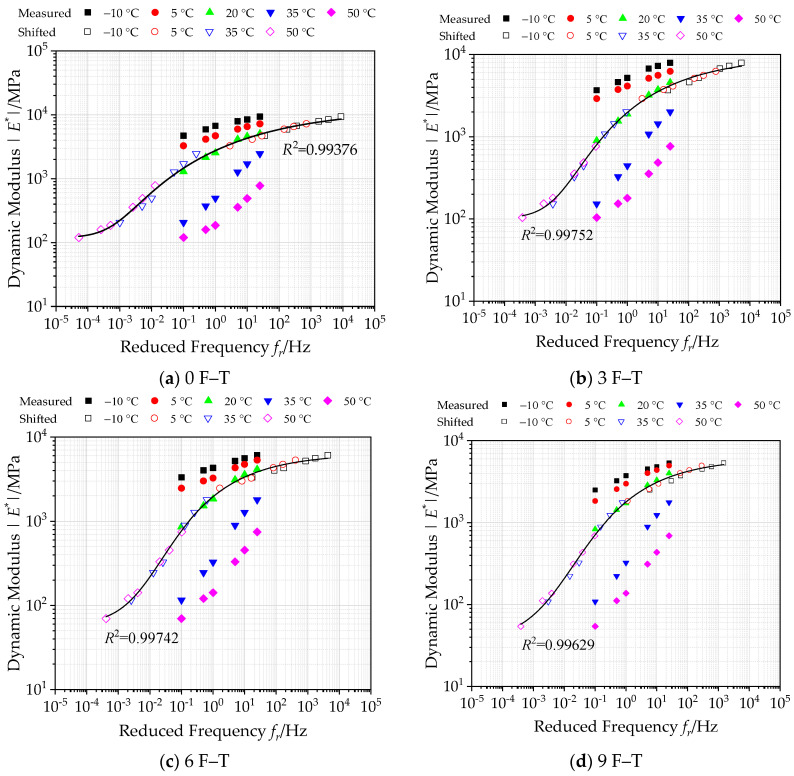

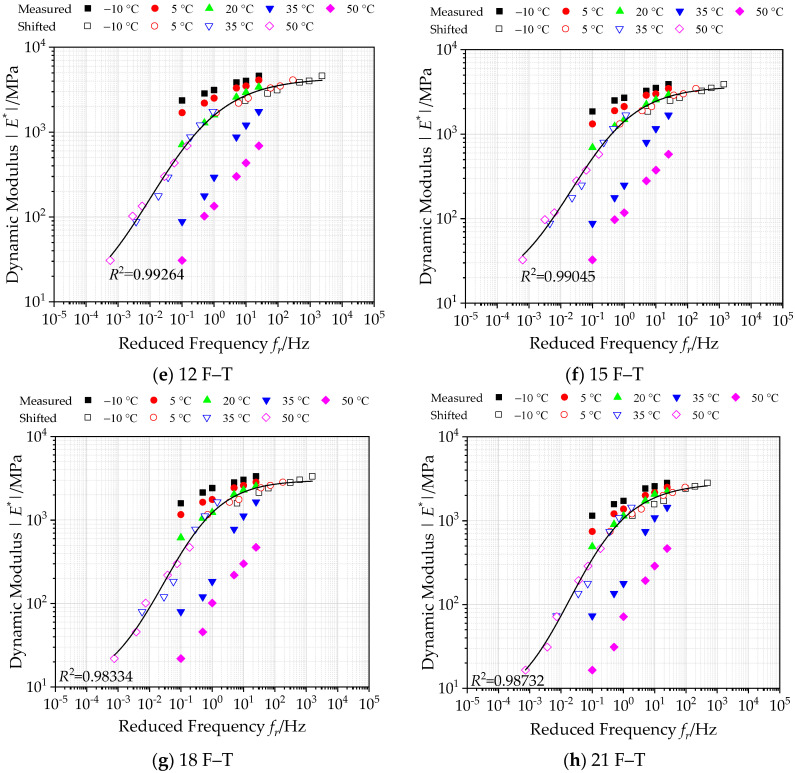
Master curves of dynamic modulus under various freeze–thaw (F–T) cycles (reference temperature = 20 °C).

On the basis of the calculated dynamic modulus |E*|, the generalized Sigmoidal function in Equation (10) is employed to establish the master curve of dynamic modulus of asphalt mixture incorporating SBS polymer and basalt fiber under different F–T actions at the reference temperature of 20 °C in a log–log graph. Figure 6 plots the calculated dynamic modulus |E*| together with the shifted measured values and the constructed master curve of dynamic modulus, and the corresponding parameters of master curve functions under various F–T cycles are listed in Table 7.

It is observed in Figure 6 that the dynamic modulus of bituminous samples under different F–T cycles always decreases gradually with increasing temperature or increases with increasing loading frequency, which shows the time–temperature correlation property of asphalt mixture. At the same time, these specimens could be ensured to stay intact while testing in the order from low temperature to high temperature and high frequency to low frequency. In addition, it could be observed that the dynamic modulus of bituminous samples gradually decreases with F–T actions increasing, which could be explained by an internal damage. Moreover, according to the summarized dynamic modulus master curves under different F–T actions in Figure 6, it can be observed that the correlation coefficient *R*^2^ values of the generalized Sigmoidal model are determined to be larger than 0.98, which verifies that the calculated dynamic modulus |E*| in the master curve can match well with the shifted data. This also demonstrates the accuracy of the master curve of dynamic modulus. The calculated master curve of dynamic modulus can extend to a wider range, which is employed to reflect the dynamic modulus of bituminous mix specimen incorporating SBS polymer and basalt fiber under different F–T actions accurately. Moreover, the calculated master curves of dynamic modulus illustrated in Figure 6 show the similar S-shaped growth trend, for which there is a changing trend of slow at both ends and fast in the middle overall; that is, its variation slows down in the high-frequency and low-frequency ranges.

#### 3.1.2. Construction of Master Curves of Phase Angle under Freeze–Thaw Cycles

**Figure 7:**
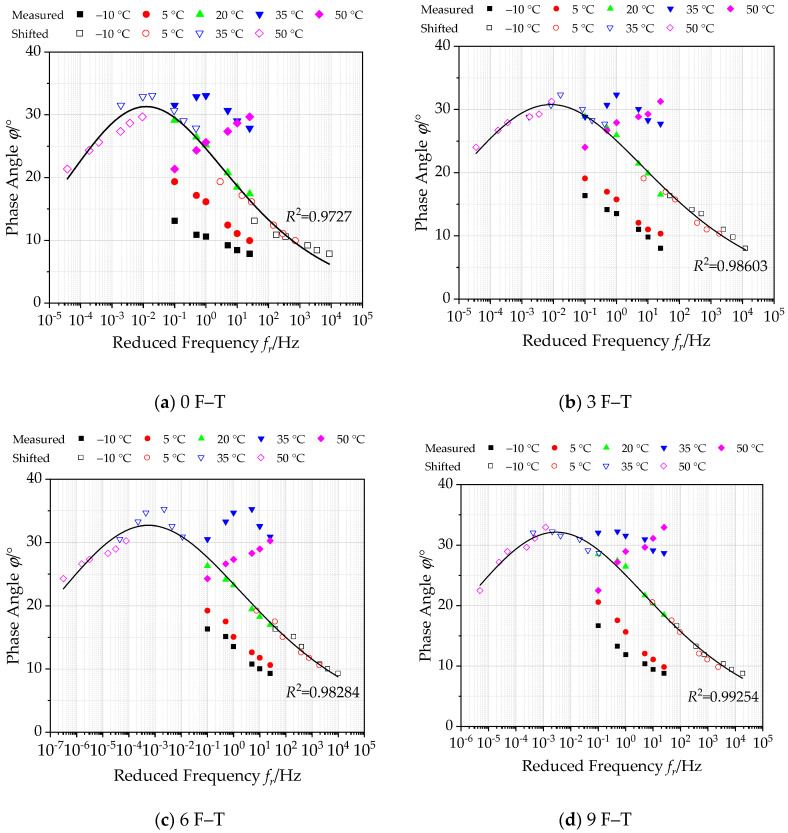

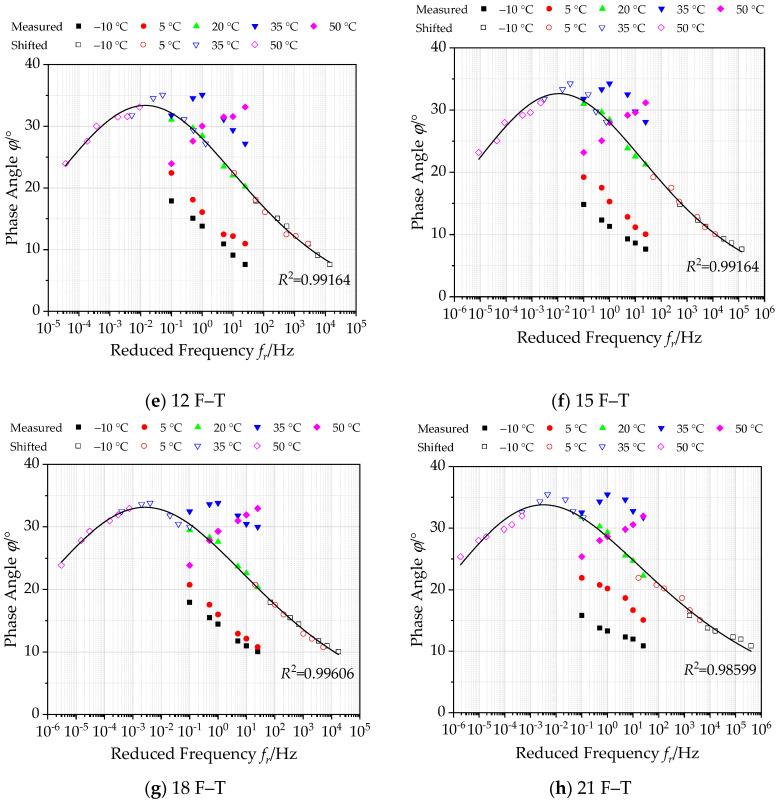
Master curves of phase angle under various F–T cycles (reference temperature = 20 °C).

The generalized Sigmoidal function in Equation (11) is employed to establish the master curve of phase angle of bituminous materials incorporating SBS polymer and basalt fiber under different F–T actions at 20 °C in a semi-log graph, as plotted in Figure 7. Figure 7 also plots the phase angle *φ* together with the shifted test data, and the corresponding parameters of master curve functions under various F–T cycles are listed in Table 8.

According to Figure 7, it can be observed that the phase angle values of bituminous mixture specimens under different F–T cycles have similar variation rules. At high temperature (35 °C and above), the phase angle exhibits an increasing trend with loading frequency. The phase angle will decrease with the increase of loading frequency if the experimental temperature is below 35 °C. This main reason for this phenomenon is that asphalt mixture mainly exhibits elastic characteristics at low temperature, and there is almost no phase angle under load. On the other hand, asphalt binder gradually softens as the test temperature increases, resulting in a larger phase angle. This is because the phase angle inside the asphalt mixture is attributed to the intrusion of aggregates when the test temperature reaches 35 °C and above. Due to aggregate being regarded as an elastic material, the phase angle will not be generated under load.

On the other hand, based on the summarized phase angle master curves under varying number of F–T actions in Figure 7, it can be observed that the correlation coefficient *R*^2^ values of the generalized Sigmoidal model are very close to 1, which verifies that the calculated phase angle φ in the master curve can match well with the shifted data. This also demonstrates the accuracy of the master curve of the phase angle. The calculated master curve of the phase angle can extend to a wider range, which is employed to reflect the phase angle of bituminous materials incorporating SBS polymer and basalt fiber under different F–T actions accurately. Besides, it is observed that the phase angle of bituminous materials increases with varying F–T actions increasing to some extent. This can be attributed to the weakening of the bond between asphalt and aggregates inside the asphalt mixture under the action of F–T. The lag phenomenon between strain and stress becomes more obvious under load.

#### 3.1.3. Dynamic Modulus Ratio of Asphalt Mixture with Basalt Fiber under Freeze–Thaw Cycles

**Figure 8:**
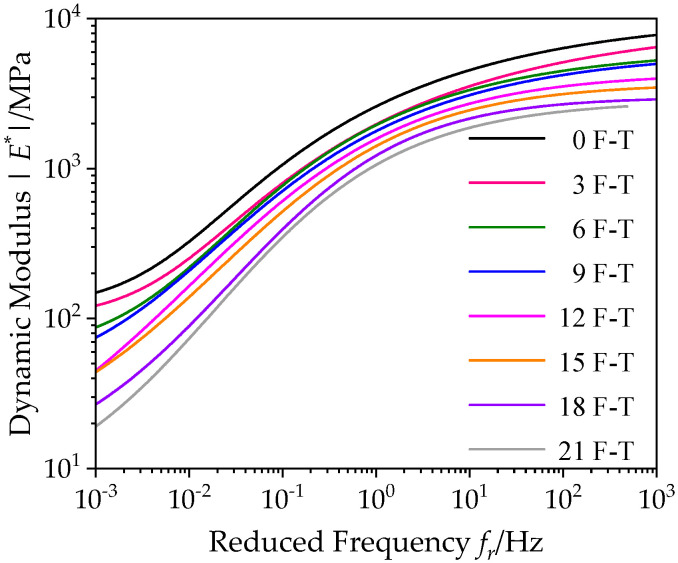
Comparison of dynamic modulus master curves under F–T cycles (20 °C).

According to the above master curves of dynamic modulus of bituminous materials incorporating SBS polymer and basalt fiber under varying number of F–T actions, Figure 8 summarizes the master curves of dynamic modulus under varying number of F–T actions in the frequency range [10^−3^, 10^3^]. By comparing these master curves, it is clearly found that the dynamic modulus of bituminous materials incorporating SBS polymer and basalt fiber gradually decreases with F–T actions, and there are differences in its changes.

In order to discuss the variation of dynamic modulus of bituminous mixes under varying F–T actions, the dynamic modulus ratio *R*_|__E__*|_ is introduced and expressed in Equation (12); that is, the ratio between dynamic modulus values |E*|*_n_* under varying F–T actions and the value |E*|_0_ before the F–T process.

(12) $$R_{\left|E^{*}\right|}=\frac{{\left|E^{*}\right|}_{n}}{{\left|E^{*}\right|}_{0}}\times 100$$

**Figure 9:**
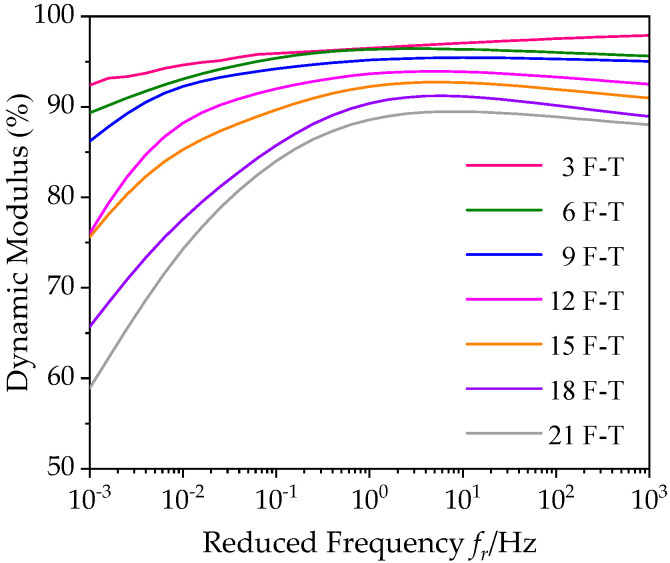
Trend of dynamic modulus ratios under F–T (reference temperature = 20 °C).

Figure 9 summaries of the dynamic modulus ratio results of bituminous material specimens under 3, 6, 9, 12, 15, 18, 21 F–T cycles at 20 °C in the frequency range [10^−3^, 10^3^]. Similarly, the dynamic modulus ratio results basically increase with reduced frequency. It could be observed that the dynamic modulus ratios of specimens are more affected by F–T cycle at a low frequency or high temperature, while the dynamic modulus of bituminous mixes change slightly at a high frequency or low temperature. This is because the actions of F–T caused the weakening of the adhesion performance between the asphalt film and aggregate, and its internal structure was damaged, but the asphalt did not suffer serious performance degradation. Therefore, asphalt mixture specimens still keep intact at low temperature, and aggregates can provide the good strength, resulting in a little change of dynamic modulus ratio. However, at high temperature, due to the destruction of bituminous mix structure, the bituminous material specimen would be relatively loose under F–T cycles, and its dynamic modulus drops sharply. Thus, in the process of road design and maintenance, attention should be paid to seasonal frozen asphalt pavement under low frequency and high temperature.

### 3.2. Influence Analysis of Freeze–Thaw Cycles on Storage Modulus and Loss Modulus of Asphalt Mixture Reinforced with Basalt Fiber

On the basis of Equations (3) and (5), the storage modulus and loss modulus can be derived by using the calculated magnitude and phase angle in Equations (8) and (9) of complex modulus

(13) $$E^{\prime }=\left|E^{*}\right|\cos\varphi$$

(14) $$E^{\prime \prime }=\left|E^{*}\right|\sin\varphi$$

where *E*’ and *E*” are the storage modulus and loss modulus, which characterize the pure elasticity and pure viscous mechanical behavior of asphalt mixtures, respectively.

Similarly, according to linear viscoelastic theory and the K-K relationship, the generalized Sigmoidal model can be adopted to construction of the master curves of the storage modulus and loss modulus of asphalt mixture, which are shown in Equations (15) and (16).

(15) $$\mathrm{lg}\left|E^{\prime }\left(f_{r}\right)\right|=\delta +\frac{\alpha -\delta }{{\left(1+\lambda \cdot e^{\beta +\gamma \mathrm{lg}f_{r}}\right)}^{\frac{1}{\lambda }}}$$

(16) $$\begin{array}{l} \mathrm{lg}\left|E^{\prime \prime }\left(f_{r}\right)\right|=\mathrm{lg}\left(-\frac{\pi }{2}\cdot \left(\alpha -\delta \right)\cdot \gamma \right)+\left(\beta +\gamma \cdot \mathrm{lg}f_{r}\right)\mathrm{lg}e\\ -\left(1+\frac{1}{\lambda }\right)\mathrm{lg}\left(1+\lambda \cdot e^{\beta +\gamma \mathrm{lg}f_{r}}\right)+\delta +\frac{\alpha -\delta }{{\left(1+\lambda \cdot e^{\beta +\gamma \mathrm{lg}f_{r}}\right)}^{\frac{1}{\lambda }}} \end{array}$$

#### 3.2.1. Construction of Master Curves of Storage Modulus under Freeze–Thaw Cycles

**Figure 10:**
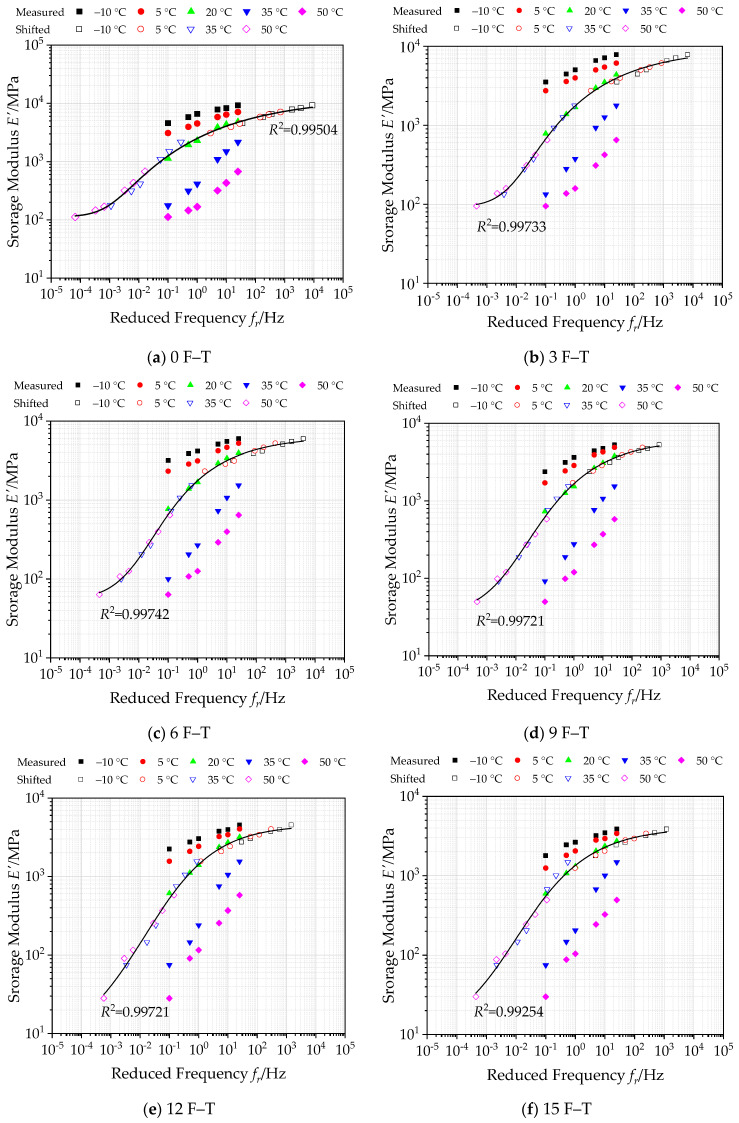

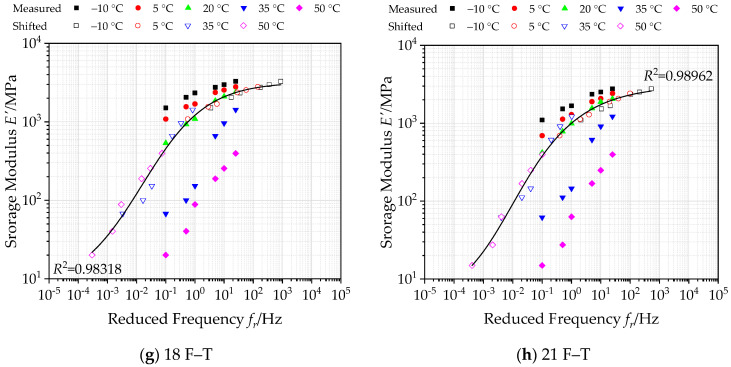
Master curves of storage modulus under various F–T cycles (reference temperature = 20 °C).

The generalized Sigmoidal function in Equation (15) is employed to construct the master curve of storage modulus of bituminous materials incorporating SBS polymer and basalt fiber under varying numbers of F–T actions at 20 °C in a log–log graph, as plotted in Figure 10. Figure 10 also plots the storage modulus *E*’ as well the shifted measured values, and the corresponding parameters of master curve functions under various F–T cycles are listed in Table 9.

As illustrated in Figure 10, the storage modulus of bituminous mixes exhibits a similar variation trend with dynamic modulus. At different loading frequencies, the storage modulus gradually decreases with increasing temperature, while the storage modulus increases with increasing loading frequency, whatever the test temperature. There is a significant change in its rate of storage modulus at a high frequency and high temperature. This could be attributed to the viscoelastic composition of asphalt mixture changing at a high frequency and high temperature, weakening the elastic behavior.

Meanwhile, it can be observed that the correlation coefficient *R*^2^ values of the summarized storage modulus master curves under different F–T cycles are very close to 1, which verifies that the storage modulus in the master curve can match well with the shifted data. The calculated master curve of storage modulus can extend to a wider range, which is employed to reflect the storage modulus of bituminous materials incorporating SBS polymer and basalt fiber under varying numbers of F–T actions accurately. Besides, the overall storage modulus of bituminous mixes decreases with F–T cycles increasing to some extent.

#### 3.2.2. Construction of Master Curves of Loss Modulus under Freeze–Thaw Cycles

**Figure 11:**
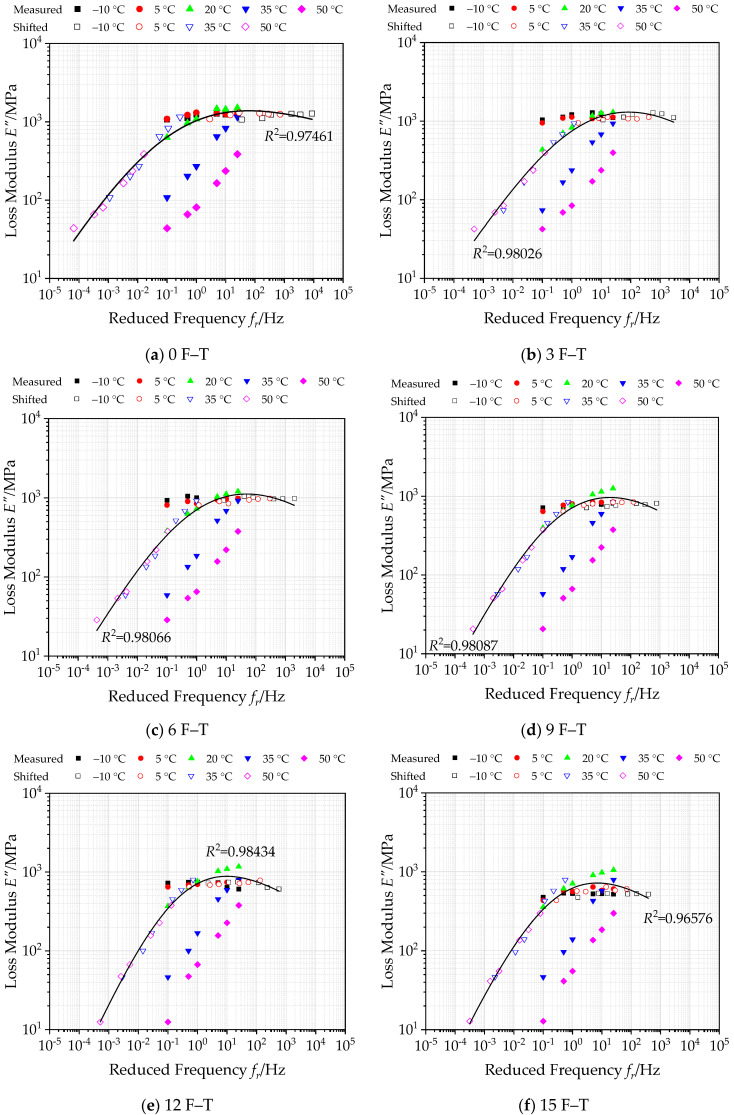

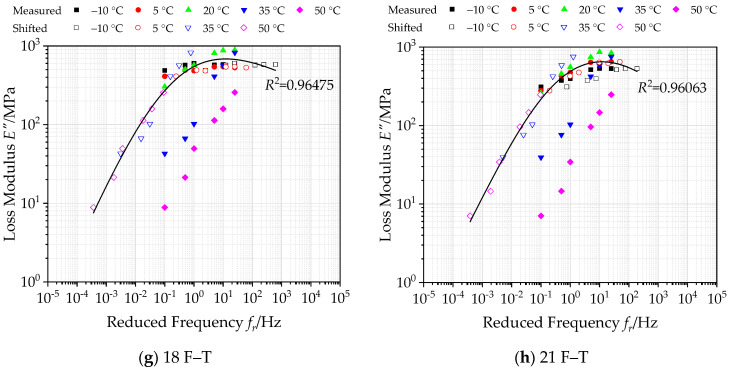
Master curves of loss modulus under various F–T cycles (reference temperature = 20 °C).

Through Equation (16), the generalized Sigmoidal function is employed to establish the master curve of loss modulus of bituminous materials incorporating SBS polymer and basalt fiber under varying F–T actions at 20 °C in a log–log graph, as well as the shifted measured values as plotted in Figure 11. The corresponding parameters of master curve functions under various F–T cycles are listed in Table 10.

Based on the loss modulus *E*” versus temperature and frequency for bituminous materials incorporating SBS polymer and basalt fiber in Figure 11, the loss modulus of bituminous mixes exhibits an increasing trend with the increasing of loading frequency at low temperatures. While at high temperature, the loss modulus increases first and then decreases with increasing loading frequency. On the other hand, it can be seen from the summarized loss modulus master curves under varying F–T actions that the correlation coefficient *R*^2^ values are very close to 1, which verifies that the loss modulus in the master curve can match well with the shifted data. The calculated master curve of loss modulus can extend to a wider range, which is employed to reflect the loss modulus of bituminous materials incorporating SBS polymer and basalt fiber under varying F–T actions accurately. Simultaneously, the overall loss modulus of bituminous mixes also decreases with F–T cycles increasing to some extent.

## 4. Conclusions

In this study, the dynamic mechanical response was analyzed for bituminous materials incorporating SBS polymer and basalt fiber. Based on the generalized Sigmoidal model, the master curves of complex modulus including dynamic modulus, phase angle, storage modulus, and loss modulus were established. Finally, the freeze–thaw evolution of bituminous materials incorporating SBS polymer and basalt fiber was evaluated by comparing the complex modulus. The main conclusions are as follows:

- As the F–T cycle increase, the dynamic modulus of bituminous materials incorporating SBS polymer and basalt fiber gradually decreases, and its phase angle shows an increasing trend. This can be attributed to the weakening of the bond between the asphalt and aggregates inside the asphalt mixture under the actions of F–T. The stress–strain lag phenomenon becomes more obvious under load.
- Based on the results of storage modulus and loss modulus, the elastic and viscous portions of bituminous materials incorporating SBS polymer and basalt fiber have decreased overall, especially significantly at low frequency and high temperature. This indicates that the performance of asphalt has been degraded to a certain extent under the actions of F–T.
- Dynamic modulus ratio results basically increase with reduced frequency. The dynamic modulus ratios of specimens are more affected by F–T cycle at a low frequency or high temperature, while the dynamic modulus of asphalt mixture specimens change slightly at a high frequency or low temperature. Thus, in the process of road design and maintenance, attention should be paid to seasonal frozen asphalt pavement under low frequency and high temperature.

## Figures and Tables

**Table 1:** Technical parameters of styrene–butadiene–styrene (SBS)-modified asphalt.

Parameters	Unit	Values
Penetration	0.1 mm (@ 25 °C, 100 g, 5 s)	72
Ductility	cm (@ 15 °C, 5 cm/min)	45
Softening point	°C	60.5
Density	g/cm^3^	1.018
Flash point	°C	262
RTFOT		
Mass loss	%	−0.094
Penetration ratio	% (@ 25 °C)	66.9

**Table 2:** Technical properties of coarse fine aggregate.

Parameters		Unit	Values	Standard Limits
Crushing value		%	13.6	≤26
Los Angeles abrasion value		%	17.9	≤28
Apparent specific gravity	13.2 mm	—	2.836	≥2.6
	9.5 mm		2.805	
	4.75 mm		2.726	
Water absorption	13.2 mm	%	0.6	≤2.0
	9.5 mm		0.28	
	4.75 mm		0.7	

**Table 3:** Technical properties of fine aggregate.

Parameters	Unit	Values	Standard Limits
Apparent specific gravity	—	2.723	≥2.5
Water absorption	%	0.64	—
Angularity (flow time)	s	39.9	≥30
Sand equivalent	%	68	≥60

**Table 4:** Technical properties of mineral filler.

Parameters		Unit	Values	Standard Limits
Apparent density		t/m^3^	2.712	≥2.5
Hydrophilic coefficient		—	0.63	<1
Water content		%	0.3	≤1
Plastic index		%	2	<4
Granular composition	<0.6 mm	%	100	100
	<0.15 mm		92.5	90~100
	<0.075 mm		81.8	75~100

**Table 5:** Technical properties of basalt fiber.

Parameters	Unit	Values
Length	mm	6
Diameter	µm	13
Specific gravity	g/cm^3^	2.55~2.65
Tensile strength	MPa	≥3000
Elongation at break	%	3.2

**Table 6:** Aggregate gradation of SMA-13.

Sieve/mm	16	13.2	9.5	4.75	2.36	1.18	0.6	0.3	0.15	0.075
Lower limit	100	90	50	20	15	14	12	10	9	8
Median (selected)	100	95	62.5	27	20.5	19	16	13	12	10
Upper limit	100	100	75	34	26	24	20	16	15	12

**Table 7:** Parameters of master curve functions of dynamic modulus under various F–T cycles.

Parameters of Master Curve	0 F–T	3 F–T	6 F–T	9 F–T	12 F–T	15 F–T	18 F–T	21 F–T
α	2.074	2.003	1.770	1.567	0.782	1.073	0.931	0.749
δ	4.098	3.980	2.802	3.787	3.638	3.568	3.477	3.455
λ	6.739	4.589	2.729	2.237	0.953	0.951	0.856	1.181
β	5.645	3.33	2.733	2.326	1.643	1.547	1.536	1.885
γ	2.352	2.13	1.683	1.356	0.827	0.924	0.990	1.092
*R* ^2^	0.99376	0.99752	0.99742	0.99629	0.99264	0.99045	0.98334	0.98732

**Table 8:** Parameters of master curve functions of phase angle under various F–T cycles.

Parameters of Master Curve	0 F–T	3 F–T	6 F–T	9 F–T	12 F–T	15 F–T	18 F–T	21 F–T
α–δ	–150.36	–175.45	–225.48	–201.93	–185.03	–204.91	–225.37	–256.73
λ	1.352	1.288	1.280	1.233	1.119	1.167	1.210	1.758
β	1.118	1.010	1.306	1.101	0.879	0.832	1.022	1.023
γ	0.587	0.486	0.401	0.434	0.476	0.426	0.398	0.411
*R* ^2^	0.97270	0.98603	0.98284	0.99254	0.99164	0.99164	0.99606	0.98599

**Table 9:** Parameters of master curve functions of storage modulus under various F–T cycles.

Parameters of Master Curve	0 F–T	3 F–T	6 F–T	9 F–T	12 F–T	15 F–T	18 F–T	21 F–T
α	–2.060	–2.009	–2.072	–2.285	–2.835	–2.612	–2.635	–2.771
δ	4.108	3.973	3.806	3.805	3.656	3.611	3.505	3.490
λ	6.619	4.492	2.707	2.112	1.011	1.441	0.961	1.647
β	5.144	3.051	2.513	2.078	1.544	1.975	1.613	2.291
γ	2.337	2.115	1.658	1.306	0.834	0.985	0.892	1.139
*R* ^2^	0.99504	0.99733	0.99742	0.99721	0.99721	0.99254	0.98318	0.98962

**Table 10:** Parameters of master curve functions of loss modulus under various F–T cycles.

Parameters of Master Curve	0 F–T	3 F–T	6 F–T	9 F–T	12 F–T	15 F–T	18 F–T	21 F–T
α	–0.017	–2.971	–3.010	–2.868	–0.003	–7.163	–0.007	–3.311
δ	9.345	4.824	4.734	4.615	10.320	11.695	9.492	4.268
λ	4.883	1.350	1.598	1.741	3.465	2.902	4.465	1.530
β	–1.085	0.388	0.542	0.699	–0.851	–0.678	–0.937	0.852
γ	0.612	0.519	0.595	0.677	0.856	0.804	0.869	0.712
*R* ^2^	0.97461	0.98026	0.98066	0.98087	0.98434	0.96576	0.96475	0.96063
